# From plausible menus to trustworthy nutrition: a risk-proportionate VALID-Diet framework for artificial intelligence-generated dietary advice

**DOI:** 10.3389/fnut.2026.1957211

**Published:** 2026-10-13

**Authors:** Jingyi Xu, Weina Xing, Lili Jia, Xi Li, Qijia Zhang, Wei Yang

**Affiliations:** 1 Department of Hepatobiliary and Pancreatic Surgery, Beijing Tsinghua Changgung Hospital, School of Clinical Medicine, Tsinghua Medicine, Tsinghua University, Beijing, China; 2 Anesthesia and Surgery Center, Beijing Tsinghua Changgung Hospital, School of Clinical Medicine, Tsinghua Medicine, Tsinghua University, Beijing, China; 3 Department of Liver ‌Intensive Care Unit, Beijing Tsinghua Changgung Hospital, School of Clinical Medicine, Tsinghua Medicine, Tsinghua University, Beijing, China

**Keywords:** dietary advice, dietetics, generative artificial intelligence, large language models, nutrition methodology, personalized nutrition, validation

## Abstract

Generative artificial intelligence can produce menus, recipes, shopping lists, and dietary explanations that appear individualized and clinically informed. Fluency alone, however, provides little evidence of nutritional accuracy, safety, feasibility, or benefit. We propose VALID-Diet, a staged framework for evaluating systems that generate actionable dietary recommendations as sociotechnical interventions. Its domains are Verification of nutritional targets and composition; Adversarial safety and boundary testing; Linkage between user inputs and recommendation changes; Implementation feasibility and inclusion; and Documentation, versioning, and reproducibility. Stage 0 defines intended use, input integrity, and a structured risk profile based on harm severity, exposure, autonomy, reversibility, and enforceable oversight. Stage 1 separates target validity, menu achievement, and arithmetic agreement and, for integrated systems, tests retrieval, calculation, and generation as distinct components. Stage 2 evaluates safety, substantive personalization, feasibility, and inclusion using pre-specified case blueprints. Stage 3 evaluates human oversight, workflow utility, and prospective outcomes only after earlier gates are passed. A retrospective worked application to a published retrieval-augmented personalized-nutrition system illustrates risk assignment and progression decisions. VALID-Diet is not a regulatory standard or consensus guideline; it is a testable proposal intended to align research claims with evidentiary strength.

## 1 Introduction

Artificial intelligence (AI) in nutrition now extends beyond food recognition and risk prediction to the direct generation of menus, recipes, shopping lists, and individualized explanations. Reviews document rapid growth in language-model applications, precision nutrition, dietary assessment, and personalized recommendation systems, alongside substantial heterogeneity in data sources, populations, evaluation methods, and intended uses (1–12). Algorithm-supported interventions can influence dietary behavior when inputs, decision rules, feedback loops, and professional roles are explicit (13, 14). Deep-phenotyping studies illustrate the biological heterogeneity that personalization may seek to address (15), while broader commentary has described both the promise and constraints of AI in nutrition research (16). None of this evidence establishes that a general-purpose generative model can safely convert an incomplete clinical profile into an actionable dietary prescription.

The methodological problem is broader than hallucinated prose. A menu may sound culturally familiar and clinically informed while its foods and portions fail to achieve the stated nutrient totals. A plan that resembles population guidance may still be unsafe for a person with chronic kidney disease, food allergy, pregnancy-related needs, medication–diet interactions, frailty, or disordered-eating risk. Generated outputs therefore need to be evaluated against the claim being made. Nutrient accuracy, personalization, safety, feasibility, professional equivalence, and health benefit are distinct claims and require distinct evidence.

The GAINS framework has advanced nutrition-specific reporting and comparability for generative-AI research (2). VALID-Diet addresses the related question of what evidence is needed before higher-risk claims or uses are considered. It places sequential gates between claims so that readability, expert preference, or user satisfaction cannot compensate for an earlier failure in nutrient computation or safety. The unit of evaluation is the complete sociotechnical system—including the model, interface, prompt logic, retrieval, calculators, food-composition sources, human review, and intended workflow—rather than the language model in isolation.

## 2 Why plausible output is not nutritional validity

Direct evaluations show why surface quality is an inadequate endpoint. Generated meal plans have displayed variable guideline concordance, energy accuracy, nutrient adequacy, reproducibility, and diet quality (17–21). In image-based assessment, food identification may be considerably better than portion-size and nutrient quantification (22, 23). Studies in dialysis and chronic kidney disease have identified clinically relevant errors in energy, protein, phosphorus, potassium, or sodium despite apparently reasonable recommendations (24, 25). Other disease-specific, weight-management, multilingual, and case-based evaluations have reached similarly mixed conclusions (26–32). Favorable expert impressions can coexist with substantial discrepancies on independent recalculation (26, 29, 32). Linguistic quality and nutritional validity should therefore be evaluated separately.

The evaluation horizon also matters. A one-day plan cannot support claims about weekly variety, recurrent inadequacy, accumulated cost, or menu fatigue. A weekly planning system needs assessment over at least one complete menu cycle. Claims about usual intake or long-term adequacy require a longer or statistically justified horizon because intake varies from day to day (33). Correlation is likewise insufficient for assessing agreement: systematic over- or underestimation may coexist with a high correlation. Signed bias and limits of agreement are more informative, but analyses must accommodate repeated or clustered observations when several days, profiles, or model runs are evaluated (34).

These distinctions form an evidentiary ladder. Generating a menu, selecting appropriate targets, achieving those targets with specified foods, responding to an individual profile, avoiding clinically important harm, performing comparably to a dietitian, and improving health are not interchangeable achievements. A fluent output may justify a bounded feasibility claim but not a safety claim. Acceptable average nutrient error may be compatible with low-risk recipe support while remaining unacceptable for unsupervised disease management. Linking each claim to the stage that supports it reduces over-interpretation and makes restricted or negative findings more informative (35–48).

## 3 Basis and scope of the proposal

VALID-Diet was developed through a structured conceptual synthesis of the empirical and methodological literature cited here. Recurrent failure modes in nutrition evaluations were considered alongside principles from nutrition methodology, medical language-model evaluation, human factors, fairness, and clinical AI deployment to identify distinct evidentiary requirements (2, 10, 11, 35–41, 49–55). This synthesis was not designed to estimate effects or exhaustively map the literature and was not a systematic review, Delphi exercise, or formal consensus process. The framework is therefore an analytically motivated, falsifiable proposal whose thresholds and progression rules require calibration, inter-rater testing, and prospective validation.

VALID-Diet comprises five domains: V, Verification of nutritional targets and composition; A, Adversarial safety and boundary testing; L, Linkage between user inputs and recommendation changes; I, Implementation feasibility and inclusion; and D, Documentation, versioning, and reproducibility. The acronym denotes domains rather than a one-to-one sequence of stages. Stage 0 defines intended use, input integrity, and a provisional risk tier; Stage 1 establishes analytical validity; Stage 2 evaluates safety and contextual validity; and Stage 3 addresses human factors, utility, and outcomes (Figure 1). Documentation is cross-cutting, and material changes trigger lifecycle monitoring and revalidation.

**Figure 1:**
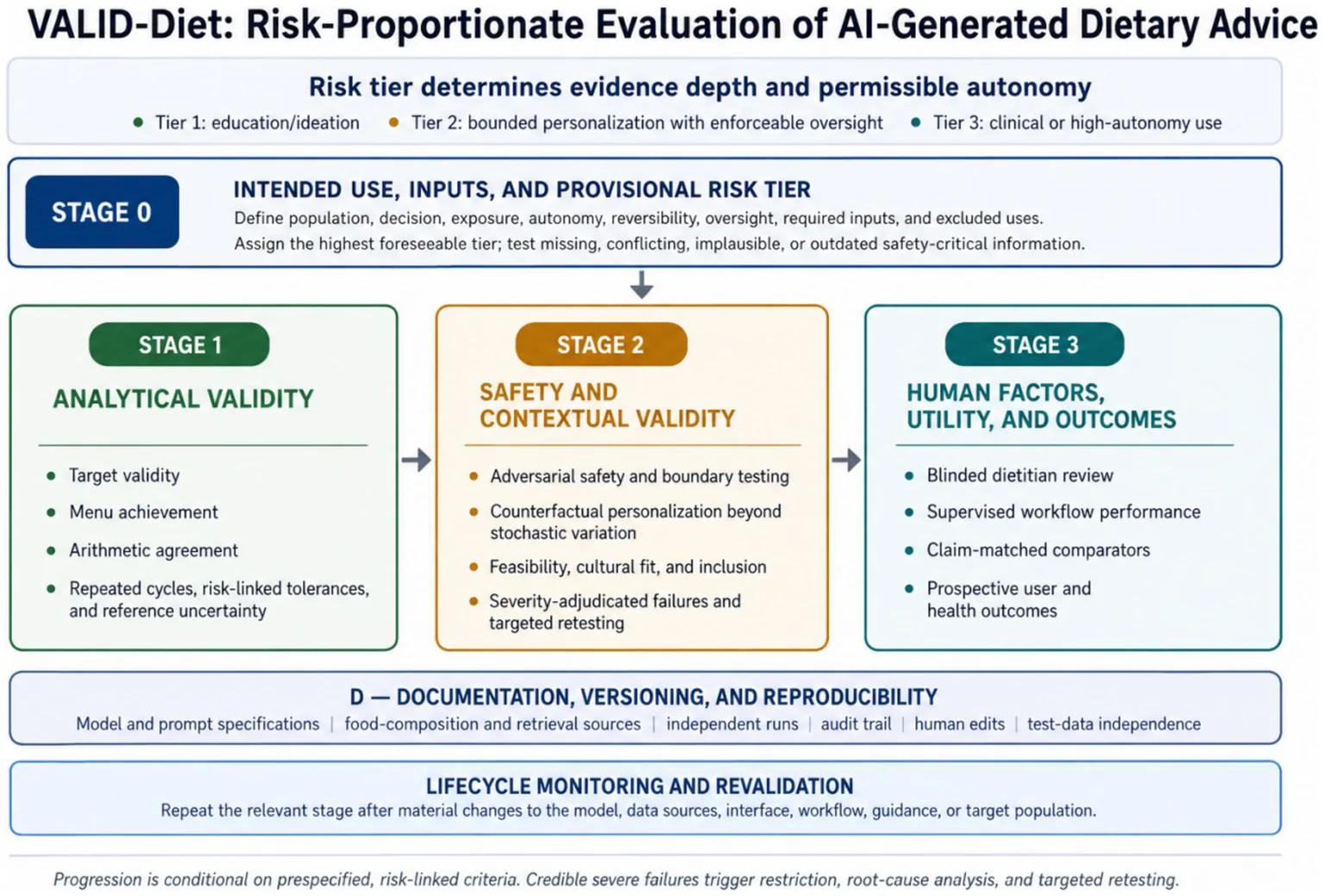
The VALID-Diet risk-proportionate evaluation pathway. Stage 0 defines intended use, input integrity, and a provisional risk tier. Stage 1 establishes analytical validity by separating target validity, menu achievement, and arithmetic agreement. Stage 2 evaluates safety, substantive personalization, feasibility, and inclusion. Stage 3 evaluates human factors, workflow utility, and prospective outcomes. Documentation, versioning, and reproducibility apply across all stages. Progression depends on pre-specified, risk-linked criteria; material changes trigger lifecycle monitoring and revalidation.

## 4 The VALID-Diet evaluation pathway

### 4.1 Stage 0: intended use, input integrity, and provisional risk tier

Evaluation begins with a precise intended-use statement: population, user, setting, decision, degree of autonomy, duration and frequency of exposure, available professional oversight, and excluded conditions. Evaluation distinguishes mandatory from optional inputs and pre-specifies behavior for missing, conflicting, implausible, or outdated information. In higher-risk settings, appropriate responses include requesting clarification, withholding an actionable recommendation, narrowing the response to low-risk education, or escalating to a qualified professional. Deliberately withholding safety-critical inputs belongs in the test design rather than an anecdotal stress test.

Risk is represented as a structured five-axis profile rather than a summed score: harm severity, exposure, autonomy, reversibility, and enforceable professional oversight (Table 1). Each axis requires an operational definition tied to observable workflow features. Assignment should draw on relevant nutrition and disease guidance, contraindications and medication–diet interactions, population vulnerabilities, prior failure data where available, and multidisciplinary hazard review (38, 41, 48). Under the proposed classification, Tier 1 covers low-risk education or recipe ideation; Tier 2 may include bounded disease-specific personalization when harm is usually reversible and prerelease professional review is mandatory and auditable; Tier 3 is proposed for uses with serious harm potential or substantial autonomy, including unsupervised disease management, medication-sensitive advice, severe allergy, advanced organ impairment, eating-disorder risk, or vulnerable life stages. Removing enforceable oversight or increasing autonomy may shift the same content toward Tier 3. At least two qualified assessors should independently assign the highest reasonably foreseeable tier and document disagreement resolution.

**Table 1:** Provisional operational VALID-diet evaluation matrix.

Stage/Domain	Minimum evaluation	Core outputs	Provisional progression rule
Stage 0: scope, inputs, and risk tier	Define the intended use and a five-axis risk profile: harm severity (minor/clinically meaningful/serious), exposure (single/episodic/repeated), autonomy (draft/prerelease-reviewed/direct-to-user), reversibility (rapidly detectable/reversible vs. delayed/difficult to reverse), and oversight (none/advisory/enforceable prerelease). Evidence may draw on workflow, clinical guidance/contraindications, medication–diet interactions, population vulnerabilities, prior failures, and multidisciplinary hazard review. Under the proposed classification, assign the highest credible Tier 1–3 pattern rather than summing points.	Intended-use statement; mandatory-input schema; dimension-by-dimension risk profile and evidence sources; tier rationale; clarification/withholding/escalation rates; inter-assessor agreement.	Do not progress if intended use, mandatory inputs, or tier are undefined, or if actionable advice is produced despite missing safety-critical information. Under the proposed classification, Tier 2 generally requires bounded use, usually reversible harm, and auditable prerelease professional review; serious harm potential or greater autonomy would generally justify Tier 3.
Stage 1: V—analytical validity	Independently assess target validity and recalculate complete menu cycles using a documented regional food-composition source. Separate target validity, menu achievement, and arithmetic agreement; pre-specify nutrient- and tier-specific tolerances. For integrated systems, test retrieval provenance/relevance/evidence linkage only as it bears on target or calculation correctness and traceability, then test calculator outputs and generation separately; context-sensitive adaptation belongs to Stage 2. Repeat sessions and account for clustered observations and comparator uncertainty.	Target concordance; menu achievement; absolute/signed error; limits of agreement where appropriate; omissions; proportion within tolerance; retrieval/evidence-linkage metrics; calculator error; between-run variation; comparator uncertainty.	Correct and repeat Stage 1 for remediable arithmetic, food-matching, or pipeline errors. Do not progress with inappropriate targets, clinically material nutrient omissions, risk-linked nutrient limits exceeded, or a non-reconstructable measurement chain.
Stage 2A: A—safety and boundaries	Use standardized case blueprints covering high-risk, conflicting-goal, adversarial, and out-of-distribution scenarios. Specify required information, hazards, prohibited recommendations, acceptable alternatives, and escalation. Test withheld safety-critical inputs and assess clarification, contraindication recognition, refusal, safer alternatives, escalation, and unnecessary refusal.	Hazard detection and unnecessary refusal; consistency; severity/reversibility/exposure-adjudicated failures; sentinel safety failures; root-cause findings; targeted-retest results.	A sentinel safety failure triggers restriction of the implicated use, root-cause analysis, and retesting. Aggregate scores cannot override the event; serious failures may require return to Stage 0 or restriction to a lower-risk scope.
Stage 2B: L + I—personalization and context	Use repeated paired counterfactual profiles and justified no-change cases; require clinically relevant effects to exceed within-profile stochastic variation. Test cost, availability, preparation burden, accessibility, language, culture, household context, and digital access.	Directional concordance; constraint satisfaction; effect beyond stochastic variation; justified invariance; repeatability; feasibility; subgroup-stratified performance.	Do not claim personalization or generalizability when relevant inputs are ignored, irrelevant attributes materially alter decision-relevant advice beyond baseline variation, or material feasibility/subgroup failures persist.
Stage 3: human factors, utility, and outcomes	Conduct blinded dietitian review and supervised workflow testing before prospective evaluation. Test oversight effectiveness: prerelease review, error detection/correction, appropriate and inappropriate overrides, disagreement resolution, escalation, review time, and correction burden. Match comparators, margins, and outcomes to the claim and tier.	Inter-rater agreement; detection/correction and override rates; unresolved disagreements; traceability; review time; correction burden; trust calibration; adherence; diet quality; adverse events; subgroup effects.	Do not infer professional equivalence or health benefit from readability, satisfaction, or benchmark accuracy. Require evidence that oversight reliably intercepts important errors and use claim-matched prospective outcomes.
Cross-cutting D and lifecycle	Document model, prompts, dates, settings, retrieval, databases, calculators, human edits, case development, test independence, and audit logs. For adaptive systems, monitor input drift, constraint violations, safety signals, and review burden. Predefine which isolated changes permit partial revalidation and which changes to decision logic, autonomy, population, guidance, or model/retrieval components repeat affected stages or the full pathway.	Reconstructable system record; version history; drift and safety signals; override/correction trends; revalidation trigger and log.	Restrict or suspend the implicated use after material drift or a safety signal. Repeat affected stages after material change; repeat the full pathway when the risk profile or clinical decision logic changes.

The tiers, thresholds, and progression rules are provisional and should be pre-specified for the intended use. Failure at an earlier gate stops progression until the cause is corrected and the affected stage is repeated. Restriction to a lower-risk use is defensible only when its boundaries and oversight are explicit and enforceable.

### 4.2 Stage 1: analytical validity

V: Verification of nutritional targets and composition—requires independent evaluation using a documented, region-appropriate food-composition source. Model-reported totals are test outputs, not validation evidence; food matching, portions, recipe decomposition, cooking yields, substitutions, unmatched items, and database provenance form part of the measurement chain (56, 57). For integrated systems, component-level checks are also needed: retrieval for source provenance, relevance, and evidence linkage; calculators against reference inputs/outputs; and the generative layer for target selection and final recommendations independently of its own intermediate values (58, 59). At Stage 1, retrieval is evaluated only for its contribution to the correctness and traceability of nutritional targets and calculations; context-sensitive adaptation belongs to Stage 2. Acceptable end-to-end output quality does not establish component validity.

Three estimands are evaluated separately. Target validity asks whether selected energy and nutrient targets are appropriate for the fully specified profile, using an explicit guideline or expert-adjudicated range. Menu achievement asks whether the listed foods and portions meet those targets. Arithmetic agreement asks whether system-reported totals agree with independent calculation. Accordingly, accurate computation does not establish validity when the underlying target is inappropriate. Stage 1 evaluates the target and realized menu for a given profile; Stage 2 tests whether clinically relevant changes in the profile produce appropriate changes in targets, constraints, and recommendations.

Agreement reporting should include absolute error, signed bias, clinically material omissions, the proportion within pre-specified tolerances, and limits of agreement where appropriate (34). Absolute and relative metrics should be pre-specified by nutrient, and clustered observations handled appropriately. Acceptance thresholds should be locked before testing by linking each nutrient to the intended decision, plausible harm, reference target, comparator uncertainty, and risk tier. Correctable arithmetic errors can be repaired and retested; an inappropriate disease-specific target or risk-linked nutrient excess blocks progression until corrected.

Testing should include repeated independent sessions and at least one complete menu cycle. Between-run variation in foods, portions, nutrient totals, refusals, safety decisions, and citations should be quantified (6, 10, 11, 49, 50). Independent recalculation is itself a measurement process: ambiguous household measures, unmatched branded foods, recipe yields, fortification, supplement formulations, and regional database differences can materially affect the comparator. A pre-specified matching protocol, documented adjudication, and reporting of substitutions or imputations are necessary; duplicate coding or sensitivity analysis is particularly useful for clinically important nutrients.

### 4.3 Stage 2: safety and contextual validity

A: Adversarial safety and boundary testing—uses standardized high-risk, conflicting-goal, and out-of-distribution cases. Core blueprints should cover allergy/celiac disease, renal or hepatic impairment, pregnancy or childhood, frailty/dysphagia/refeeding risk, diabetes therapies and anticoagulants, eating-disorder signals, unsafe weight-loss requests, and competing objectives. Each blueprint specifies required information, hazards, prohibited recommendations, acceptable alternatives, and escalation criteria. Evaluation records clarification seeking, contraindication recognition, uncertainty, refusal, safer alternatives, and escalation (35–38, 51–55).

Case-level failures require structured adjudication of harm severity, reversibility, exposure, and plausibility of the causal pathway. A credible severe failure without effective mitigation is a sentinel safety failure, prompting root-cause analysis, temporary restriction of the implicated use, and targeted retesting rather than averaging it away. Conversely, unnecessary refusal should be reported because it can shift work and restrict access. Correctable performance errors can return to the affected gate; serious safety failures can require scope restriction or reassignment at Stage 0.

L: Linkage between user inputs and recommendation changes—treats personalization as a falsifiable property. Paired counterfactual profiles hold most inputs constant while changing one clinically or practically relevant variable, such as kidney function, pregnancy status, allergy, medication, budget, cooking facilities, religious practice, or activity level. The expected result is not one uniquely correct menu but an expert-adjudicated directional change in targets, constrained foods, portions, timing, monitoring, or safety advice. No-change cases should be restricted to attributes pre-specified as irrelevant under an explicit clinical or causal rationale.

Because generative systems are stochastic, each baseline and counterfactual profile should be tested in repeated, independent, order-randomized sessions under the same configuration. Counterfactual effects should exceed within-profile between-run variation before they are attributed to personalization. Clinically irrelevant attributes may change surface choices such as recipe selection; failure occurs when they materially change targets, safety constraints, or other decision-relevant recommendations beyond baseline variation. Useful outputs include directional concordance, constraint satisfaction, clinically material change, justified invariance, and repeatability.

I: Implementation feasibility and inclusion—asks whether a complete plan can be understood, afforded, obtained, prepared, and followed in the intended food environment. Evaluation should address local availability, cost, preparation time, equipment, food and digital literacy, accessibility, language, household structure, customary eating patterns, and religious practice. Subgroup results should not be averaged away because medical AI can reproduce demographic and race-based assumptions (60, 61). Cultural adaptation is stronger when co-designed with intended communities; translation or token food substitution is not equivalent to cultural safety (62, 63). Usability and engagement also vary by population and intervention design (64).

Safety, personalization, and feasibility benchmarks are best specified as case blueprints rather than loosely worded prompts. Dietitians, relevant clinicians, human-factors specialists, and affected communities should contribute to case development. Adjudicators should score observable behavior, preserve ratings before consensus, report inter-rater agreement, and present severe individual failures separately from aggregate performance (37–41, 60–63, 65, 66).

### 4.4 Stage 3: human factors, clinical utility, and outcomes

Passing analytical and contextual gates does not establish professional equivalence or benefit. Stage 3 begins with blinded review by qualified dietitians using separate ratings for linguistic quality, nutritional adequacy, clinical safety, feasibility, and actionability. Oversight itself should be tested: studies should document prerelease review, final accountability, detection and correction of unsafe or inaccurate outputs, appropriate and inappropriate overrides, unresolved disagreement, escalation, review time, and correction burden (38–41, 67, 68). Assessor qualifications, training, arbitration, and inter-rater agreement should be pre-specified.

Prospective outcomes should match the claim. Low-risk meal-planning aids may be evaluated for comprehension, adherence, dietary variety, cost, and preparation burden. Disease-specific systems may additionally require validated diet-quality indices, symptoms, laboratory measures, body composition, medication-related safety events, or other clinical endpoints. Benchmark accuracy, satisfaction, and short-term engagement do not demonstrate improved health. Efficiency requires measurement of professional time and correction burden; equivalence to dietitian care requires a qualified comparator, blinded assessment, and clinically meaningful equivalence margins; benefit requires prospective comparison with an appropriate control (69, 70).

Where ethically permissible, studies could compare model-only, professional-only, and professional-plus-model conditions. Outcomes should distinguish errors introduced by the system, errors intercepted by reviewers, and correct recommendations that reviewers inappropriately override. Human oversight can reduce risk but can also conceal model weakness or introduce automation bias. Time savings are therefore interpretable only alongside correction burden, confidence calibration, and residual risk.

### 4.5 Retrospective worked application to a published personalized-nutrition system

To illustrate practical use without new experimentation, we retrospectively mapped evidence reported by Gavai and van Hillegersberg for a RAG-based system for obesity and type 2 diabetes onto VALID-Diet (58). Among 1,000 generated smoothie recipes, 80.1% met the study’s nutritional criteria and 92% its sustainability criteria. Under the proposed classification, mandatory prerelease dietitian review could support Tier 2 use, whereas direct-to-user disease management or greater autonomy would generally justify Tier 3. These internal validation results provide partial evidence toward menu achievement but do not establish Stage 1 because target validity and arithmetic agreement were not independently verified. Stage 2 is not established because paired counterfactual testing and a pre-specified severe-harm battery were not reported; Stage 3 is unsupported because professional workflow and clinical outcomes were not evaluated. This retrospective mapping shows that conclusions should stop at the strongest supported gate: RAG or internal validation cannot replace independent nutritional verification, and a sentinel safety failure would require restriction, root-cause analysis, and retesting.

## 5 Documentation, versioning, and reproducibility across the lifecycle

D applies across all stages. A reconstructable report identifies the provider, model and version, access dates, training-data cutoff when known, system instructions, complete prompts, conversation history, sampling settings, retrieval sources, calculators, food-composition database and version, post-processing, and human edits. It should also document prompt development, test-data independence, session structure, case adjudication, and material software or workflow dependencies. Reviews indicate that stochasticity, exact prompts, query dates, and test independence are often omitted from health-language-model studies (37, 45–47).

Versioning has direct safety implications. Static systems require revalidation after material changes; adaptive systems that update recommendations from new user data, feedback, retrieved evidence, or model behavior additionally require ongoing monitoring of input distributions, constraint violations, safety signals, and review burden. Partial revalidation is defensible only when a change is isolated and cannot alter clinical decision logic. Changes to model or prompt policy, target equations, food-composition sources, clinical guidance, retrieval logic/corpus, autonomy, safety rules, workflow, or target population warrant repetition of affected stages and may require the full pathway if the risk profile changes (48, 59, 71, 72). Temporal instability should be treated as a performance characteristic rather than noise (73, 74).

## 6 Progression rules and relation to existing guidance

Table 1 translates VALID-Diet into minimum evaluations, core outputs, and provisional progression rules. We do not propose a universal nutrient-error cutoff or aggregate score. Acceptable error depends on the nutrient, population, decision, exposure, comparator uncertainty, and effective review. Investigators should pre-specify decision-specific thresholds. Undefined intended use, actionable advice despite missing safety-critical information, a sentinel safety failure, or an unreconstructable system are candidate hard stops. Correctable analytical errors may be repaired and retested; safety failures may require scope restriction, return to Stage 0, or suspension. These rules remain hypotheses to be calibrated.

VALID-Diet complements rather than replaces existing guidance. GAINS supports nutrition-specific reporting and comparability (2); DECIDE-AI addresses early clinical evaluation; TRIPOD+AI/TRIPOD-LLM and MI-CLEAR-LLM address reporting; FUTURE-AI addresses trustworthy deployment; and SPIRIT-AI/CONSORT-AI address AI intervention protocols and reports (42–48, 75). VALID-Diet adds nutrition-specific operational requirements for actionable generation: sequential evidentiary gates, independent verification of targets and realized composition, counterfactual personalization tests, case-level safety sentinels, and risk-linked progression criteria. It is therefore an evidence pathway, not a replacement reporting standard.

## 7 Research priorities and limitations

An initial empirical validation of VALID-Diet could use standardized case sets spanning chronic diseases, life stages, languages, cultural dietary patterns, and resource settings. Independent dietitians and relevant clinicians should rate tier assignment, clinically important nutritional errors, severe safety failures, counterfactual personalization, and progression decisions across several AI systems and oversight models; inter-rater agreement should be reported before consensus. Replication across food-composition databases and protected test cases would test generalizability while reducing benchmark contamination (37–48).

VALID-Diet remains a proposal. Its risk tiers and progression thresholds have not been empirically calibrated or prospectively validated across populations and health systems. A severe-failure rule may be appropriately conservative for Tier 3 use yet unstable in small test sets. Independent nutrient calculation improves accountability but cannot eliminate food-composition uncertainty. Professional oversight may reduce harm while masking model deficiencies and increasing workload. Cultural fit, acceptable personalization, and severity of potential harm require structured judgment rather than one automated score. Multi-stakeholder consensus work, inter-rater studies, benchmark challenges, and prospective implementation research are therefore priorities.

## 8 Discussion

Generative AI may widen access to food ideas, reduce the burden of drafting menus, support multilingual communication, and help dietitians explain complex recommendations. These possibilities deserve rigorous testing. Nutrition is also unusually vulnerable to an illusion of correctness: a quantitative or clinical error can recur across meals, interact with disease or medication, and remain unnoticed because the output is familiar and well written.

The central methodological point is that evidentiary demands should match the consequences of error. Analytical performance may support a limited claim for low-risk recipe assistance, whereas disease-specific or autonomous recommendations require stronger evidence on safety, personalization, oversight, and outcomes. Intended use and a documented risk profile therefore come first, followed by analytical validity, contextual safety, and, when justified, workflow and outcome evaluation. The worked application illustrates why strong performance at one layer does not validate untested later claims. VALID-Diet will be useful only if its gates are calibrated and revised through empirical testing.

## Data Availability

The original contributions presented in the study are included in the article/supplementary material, further inquiries can be directed to the corresponding authors.
